# Intramural cyst originating from Luschka’s duct in the gall bladder: A case report

**DOI:** 10.1016/j.ijscr.2021.105794

**Published:** 2021-03-23

**Authors:** Shiori Saeki, Johji Imura, Tadashi Bando, Kazuto Shibuya, Isaku Yoshioka, Tsutomu Fujii

**Affiliations:** a Department of Surgery and Science, Faculty of Medicine, University of Toyama, 2630 Sugitani, Toyama City, Toyama, 930-0194, Japan; b Department of Diagnostic Pathology, Faculty of Medicine, University of Toyama, 2630 Sugitani, Toyama City, Toyama, 930-0194, Japan; c Department of Surgery, Toyama Saiseikai Hospital, 33-1 Kusunoki, Toyama City, Toyama, 931-8533, Japan

**Keywords:** RAS, Rokitansky-Aschoff sinus, CEA, carcinoembryonic antigen, CA, carbohydrate antigen, CT, computed tomography, DIC, drip infusion Cholecystocholangiography, CK, cytokeratin, αSMA, α-smooth muscle actin, Case report, Gallbladder, Intramural cysts, Luschka’s duct

## Abstract

**Introduction and importance:**

An intramural cyst is a rare lesion that develops in the wall of the gallbladder. Although the acquired cysts originate from the Rokitansky-Aschoff sinus (RAS), the congenital them, such as the duct of Luschka, are rare. Luschka's duct is a unique and specific tissue component that is histologically different from the inherent bile duct and without the communication to the lumen of the gallbladder.

**Case presentation:**

A woman in her seventies underwent cholecystectomy for the treatment of repeated choledocholithiasis. Pathological examination of the resected specimen revealed multiple cysts in the subserosal tissue of the liver bed. The cysts were lined by cuboidal epithelium and surrounded by hypercellular fibrous tissue. Apart from the Luschka's ducts scattered around the cyst, no other components were observed. Immunohistochemically, the cystic epithelium was different from that of the gallbladder and phenotypically similar to that of the Luschka's duct.

**Discussion:**

From histopathological and immunohistological findings, it was suggested that the cysts of the present case are not derived from RAS, which is the most common in the gallbladder, but from the Luschka’s duct.

**Conclusion:**

We report an extremely rare case of intramural cysts that appear to have originated from the Luschka’s duct.

## Highlights

• A case of the intramural cyst of the gallbladder is rarely encountered.
• The cyst derived from the Luschka duct, a specific histological element of the gallbladder, has not received much attention.
• The gallbladder’s mural cyst, which is derived from the Luschka duct, is different from Rokitansky-Aschoff sinus origin.

## 1 Introduction

Reports of intramural cysts in the gallbladder are rare; only a few cases have been reported since it was first described by Wiedman [1]. Various hypotheses have been proposed for the development congenital, acquired, and neoplastic cysts have been reported in the literature. Most of them are derived from the Rokitansky-Aschoff sinus (RAS) due to mucosal invagination into the wall and loss of communication with the lumen of the gall bladder, as seen in diverticulosis [2]. Other reports have indicated the development of cysts from tissues such as the rests of fetal components [3], the aberration of the gastric mucosa [4]; other examples include the epidermoid [5], mesothelial [6], and foregut cysts [7] and the duct of Luschka [8,9]. To the best of our knowledge, only two cases of the duct of Luschka have been reported so far. However, they have not been proven to originate from Luschka’s ducts via objective methods. Herein, we present an extremely rare case of an intramural cyst in the gallbladder. Histopathological and immunohistochemical studies were used to confirm that the cyst originated from the Luschka’s duct.

## 2 Case presentation

**Fig. 1:**
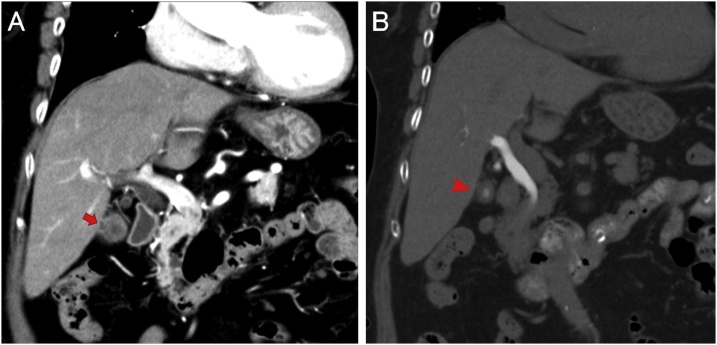
Radiological findings. (A) A cystic lesion (red arrow) was noted in the wall of the gallbladder via enhanced abdominal computed tomography (CT). (B) The cyst was not enhanced by drip infusion cholecystocholangiography-CT (red arrowhead).

**Fig. 2:**
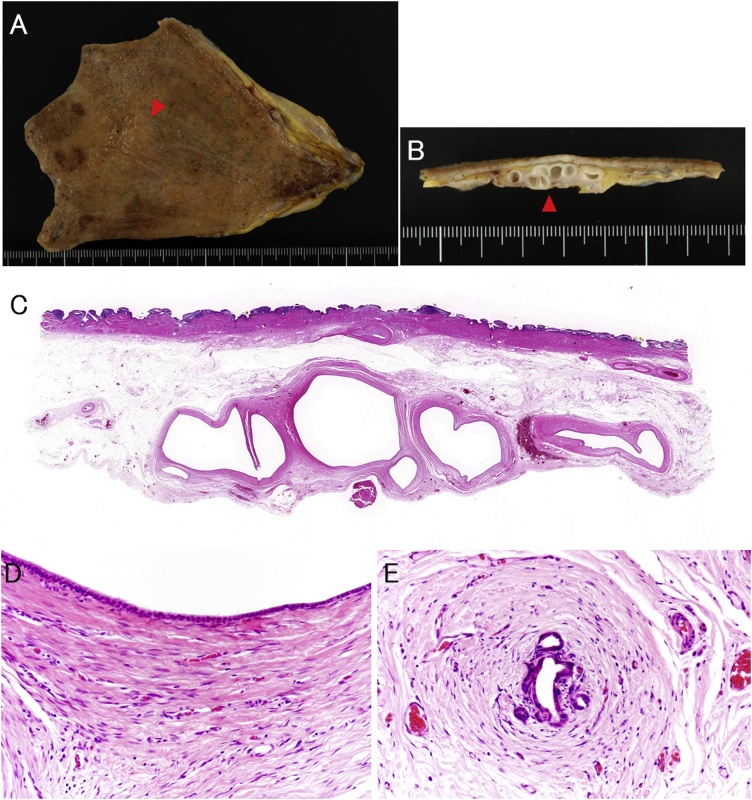
Macroscopic and histopathological findings. (A) On the inner surface of the gallbladder, a slightly elevated lesion was observed on the liver bed side at the bottom (red arrow). (B) On the cut surface, several cysts were seen within the wall of the elevated lesion (red arrowhead). (C) Several well-defined bordered cysts were seen in the subserosal layer only. (D) The cyst lining consisted of a monolayer of cuboidal cells. Dense and cellular fibrous tissue encircled the cyst. (E) Small structures suggestive of Luschka’s duct were scattered around the cyst.

**Fig. 3:**
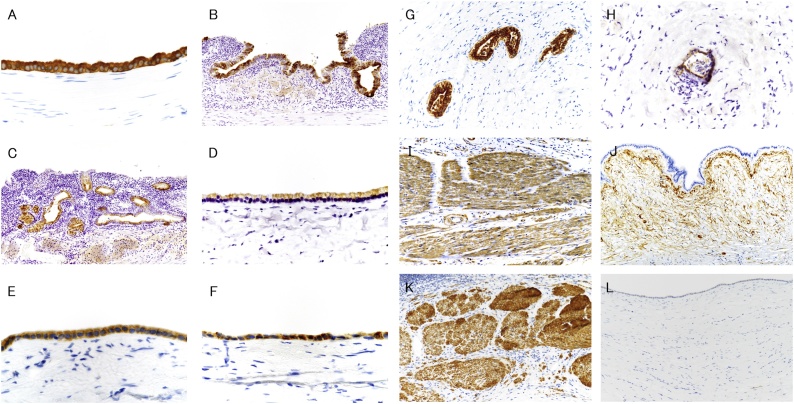
Immunohistochemical findings. (A and G) CK7, (B) MUC5AC, (C, D and H) MUC6, (E) MUC2, (F) CEA, (I and J) α-smooth muscle actin (αSMA), and (K, L) desmin. Cytoplasmic expression was observed in the cystic epithelium (A, D, E, F) and Luschka’s duct epithelium (G, H). Cytoplasmic expression was observed in the mucosal epithelium (B, C). Dense cellular fibrous tissue around the cyst and fibromuscular layer showed a positive reaction for αSMA (I and J), but desmin was expressed only in the fibromuscular layer (K) and not in the cyst wall.

The patient was a woman in her seventies and had a history of appendectomy, hypothyroidism, and cataracts. She had undergone endoscopic papillary balloon dilatation for choledocholithiasis about 2 years ago. Thereafter, the choledocholithiasis recurred and endoscopic sphincterotomy was performed. Subsequently, the patient visited Saiseikai Toyama Hospital for surgery due to symptoms of recurrence. No findings of jaundice or anemia were noted; the abdomen was flat and soft without tenderness and peristaltic sounds of the bowel were regular. Laboratory findings revealed no inflammation or increases in the levels of the hepatobiliary enzymes. The levels of the tumor markers carcinoembryonic antigen (CEA) and carbohydrate antigen (CA) 19-9 were within the normal ranges. Abdominal computed tomography (CT) showed a low absorption zone in the gallbladder wall suggestive of a cyst. The low absorption areas displayed lower CT values than the gallbladder lumen (Fig. 1A). In addition, the presence of stones (about 15 mm in diameter) in the gallbladder was detected by drip infusion cholecystocholangiography-CT (DIC-CT) imaging. No contrast medium was observed in the suspected cyst area (Fig. 1B). Abdominal ultrasonography revealed the presence of bile sludge, but no abnormalities in the gallbladder wall other than the cysts and the stone were noted. Laparoscopic cholecystectomy was performed and the patient was diagnosed with cholecystocholedocholithiasis. No adhesions in the surrounding tissues of the gallbladder were observed during intraoperative examination. A whitish region different from the original color of the wall was observed at the boundary between the liver bed and the bottom of the gallbladder when the gallbladder was stripped from the liver. The gallbladder was carefully resected to include the whitish region. Macroscopically, apart from the biliary sludge, no stones were observed in the gallbladder. The mucosal surface of the gallbladder maintained a normal reticular pattern, and no ulcerated or depressed lesions were observed. However, a slightly elevated lesion was observed on the liver bed side at the bottom of the gallbladder, which appeared like a white-colored submucosal tumor (Fig. 2A). Although no thickening of the gallbladder wall was observed on the cut surface, several cysts were found within the wall corresponding to the elevated lesion on the liver bed side (Fig. 2B). The lumen of the cyst contained a transparent serous liquid with no bile juice, biliary sludge or stones. Histopathologically, several cysts with diameters ranging from 3 to 5 mm were found in the subserosal layer on the hepatic side of the liver bed (Fig. 2C). The lining epithelium on the inner surface of the cyst, although partially flattened, consisted of a monolayer of cuboidal or low columnar cells, which did not demonstrate atypia. The nuclei of the cells were small and round to oval in shape, and the cytoplasm was clear or weakly eosinophilic. No cilia were found in the lining epithelium. Only exfoliated epithelial pieces were observed floating in the cyst lumen. The cysts were encircled by dense fibrous tissue intermingled with the fibroblastic cells (Fig. 2D). However, no muscle fibers like those existing in the fibromuscular layer of the gallbladder were observed around the cyst. Furthermore, a muscle fiber layer reminiscent of the muscularis propria of the intestinal tract was absent. The small tubules were surrounded by concentric dense fibrous tissue and comprised of narrow lumens lined by low columnar epithelia; these were thought to be the ducts of Luschka (Fig. 2E). Lymphocytic infiltration was observed in the existing lamina propria, but no hyperplastic changes, invagination of the mucosal epithelium or RAS were detected. The immunohistochemical results are shown in Table 1. The cyst lining epithelium and mucosal epithelium were positive for cytokeratin 7 (Fig. 3A) and negative for cytokeratin 20 (CK20) and CDX-2. A positive reaction for CD10 was observed on the luminal surface side. In the mucous phenotype, MUC5AC was strongly positive in the cytoplasm of the mucosal epithelium (Fig. 3B) but negative in the cystic epithelium. Although positive immunoreactions for MUC6 were observed in both types of epithelia, only low columnar cells on the crypt bottom of the mucosal epithelium demonstrated partial positive reactions (Fig. 3C), whereas in the cystic epithelium, scanty positive reactions were observed in the cytoplasm of the cells (Fig. 3D). Positive reactions for MUC2 (Fig. 3E) and CEA (Fig. 3F) were observed only in the cystic epithelium. However, podoplanin and CD34 were negative in both the epithelia. In addition, the coexisting Luschka’s duct epithelium showed the same expression pattern as that in the cystic epithelium (Fig. 3G, H). Furthermore, the fibroblastic cells and fibrous components around the cyst and the Luschka’s duct, as well as the smooth muscle cells and fibers that constituted the fibromuscular layer in the gallbladder wall, were all positive for α-smooth muscle actin (αSMA; Fig. 3I, J). Nonetheless, immunoreactions for desmin were positive only in the fibromuscular layer of the gallbladder (Fig. 3K) and negative in the cyst wall (Fig. 3L).

**Table 1:** Results of immunohistochemical studies.

		Epithelium	
Antibody (clone, source)	Cyst	Luschka's duct	Mucosa
Cytokeratin 7 (SP53, Roche)	+++	+++	+++
Cytokeratin 19 (A53B/A2.26, Roche)	+++	+++	+++
Cytokeratin 20 (SP33, Roche)	−	−	−
CD10 (SP67, Roche)	+*	+*	+*
CDX-2 (AMT28, Leica)	−	−	−
MUC1 (H23, Roche)	+	+	+
MUC2 (MRQ-18, Roche)	+	−	−
MUC5AC (AMT28, Novocastra)	−	−	++
MUC6 (CLH5, Novocastra)	+	+	++
CEA (TF3H8-1, Roche)	++	−	−
CA19-9 (121SLE, Roche)	+++	+++	+++
Podoplanin (D2-40, Roche)	−	−	−
CD34 (QBEnd/10, Roche)	−	−	−
−: negative, +: week cytoplasmic positive, ++: strong cytoplasmic positive. +++: strong membranous and cytoplasmic positive, +*: luminal surface positive.			

	Surrounding cell and fiber		
Antibody (clone, source)	Cyst	Luschka's duct	Fibromuscular layer
Actin, Smooth Muscle (1A4, Roche)	+	+	+
Desmin (DE-R-11, Roche)	−	−	+

−: negative, +: positive.

Based on these histopathological and immunohistochemical findings, the patient was finally diagnosed with an intramural cyst of the gallbladder derived from the duct of Luschka.

## 3 Discussion

This report presents a differential diagnosis for cysts that form in the wall of the gallbladder. Among the various types of cysts, including congenital, acquired, and neoplastic cysts, the acquired types are the most frequently observed and are commonly known as RAS-derived cysts. These cysts are formed due to the expanding of the invaginated mucosa within the wall, and the subsequent loss of continuity with the lumen. The presence of the RAS around the intramural cyst and the condensed bile within the cyst lumen support these inferences. Further evidence is provided by the presence of tall columnar cells in the inner lining of the epithelium of the cyst, similar to the mucosal epithelium in the gallbladder.

The current case was not considered as a RAS-derived cyst because no RAS lesions were observed around the cysts, which were located only in the subserosal layer. Furthermore, the hypercellular fibrous tissue was different from the lamina propria that surrounded the cysts. In the DIC-CT images, the cysts were not enhanced by contrast medium, thus indicating that there was no direct communication between the cyst and the mucosal lumen of the gallbladder. Congenital cysts include the cyst-hepatic duct rests of the fetal components [3], aberrations of gastric mucosa [4], epidermoid cysts [5], mesothelial cysts [6], and foregut cysts [7]. The cysts are synchronously present in the specimen, and no other gland components or supporting findings are observed. The lining epithelia of the cysts present with characteristic features; dermoid cysts consist of stratified squamous epithelium with keratinization, whereas mesothelial cysts consist of cuboidal mesothelial cells. The lining epithelium of the foregut cyst is composed of ciliated pseudostratified cells and a muscularis propria layer [7]. In the present case, no cilia or muscularis propria specific to foregut cysts were found.

According to Cureton and Newcombe, the cyst derived from Luschka’s duct can be identified based on four features: 1. The location of the cyst between the liver and the gallbladder on the liver bed; 2. The presence of a small duct similar to the Luschka's duct present around the cyst; 3. The presence of a lower lining epithelium, unlike the tall mucosal epithelium of the gallbladder; and 4. No communication with the gallbladder lumen and other biliary tracts [8]. The cysts in the present case report were located in the subserosal connective tissue on the hepatic side of the gallbladder. The cyst wall contained no elements of the lamina propria or fibromuscular tissue, and the Luschka's ducts were scattered around the cysts.

The immunophenotypes of the cystic epithelium, Luschka’s duct epithelium, and mucosal epithelium are similar. However, the cystic epithelium was partly different from the mucosal epithelium and showed an expression pattern similar to that of the Luschka’s duct in the current study. MUC5AC was expressed only in the mucosal epithelium; MUC6 was strongly expressed in the mucosal epithelium and weakly in the cystic epithelium was weakly. In addition, unlike the fibromuscular layer of the gallbladder, the hypercellular fibrous tissue around the cyst showed negative staining for desmin and positive staining for αSMA.

Luschka's duct is a small bile duct with a diameter of 1–2 mm located between the gallbladder and the liver parenchyma. It drains various areas of the right posterior segment of the liver and is thought to mostly flow into the right hepatic duct or common bile duct. There is no communication with the lumen of the gallbladder [10]. Although its role is unknown, it is considered to be a unique duct that exists around the subserosal layer of the gallbladder. Luschka's duct exhibits a unique tissue architecture and shows a different immunophenotype from the biliary tract epithelium [11].

MUC2 was positive only in the cystic epithelium. MUC2, CK20, and CDX2 are known as markers of goblet cells in the intestinal mucosa. During childhood, the bile tract mucosa contains goblet cells, many of which are positive for MUC2, CK20, and CDX2 at birth. These cells seem to decrease in number with age. Furthermore, intestinal metaplasia of the gallbladder epithelium may represent the phenotype of the immature bile duct epithelium [12]. The cystic epithelium may present with an immature phenotype as a result of the dilation of the Luschka's duct. Similarly, CEA is not originally expressed in Luschka's duct, and is positively expressed only in the cystic epithelium. This expression may have been acquired in response to cyst formation and the neoplastic transformation.

## 4 Conclusions

The cysts originating from the Luschka's duct are considered to be extremely rare. The cases reported as intramural cysts of the gallbladder might have been mistakenly diagnosed as RAS or cysts derived from RAS. Thus, such cysts should be carefully observed in order to reach a proper diagnosis.

## Declaration of Competing Interest

The authors report no declaration of interest.

## Source of funding

There were no external funding sources for this work.

## Ethical approval

This case report followed the guidelines of the Ethics Committee of Saiseikai Toyama Hospital (Number Rin 2 – 7 – 30 - 2).

## Consent

Oral consent has been obtained from the patient for the publication of this case report with accompanying images.

## Author contributions

SS and JI were responsible for the acquisition and interpretation of patient data and manuscript preparation. TB, KS, IY and TF participated in the interpretation of patient clinical data. SS, JI and AN performed the pathological examination. All authors approved the ﬁnal manuscript.

## Registration of research studies

Not applicable.

## Guarantor

Johji Imura.

## Provenance and peer review

Not commissioned, externally peer-reviewed.
